# ADCY5-related movement disorders: Frequency, disease course and phenotypic variability in a cohort of paediatric patients

**DOI:** 10.1016/j.parkreldis.2017.05.004

**Published:** 2017-08

**Authors:** Miryam Carecchio, Niccolò E. Mencacci, Alessandro Iodice, Roser Pons, Celeste Panteghini, Giovanna Zorzi, Federica Zibordi, Anastasios Bonakis, Argyris Dinopoulos, Joseph Jankovic, Leonidas Stefanis, Kailash P. Bhatia, Valentina Monti, Lea R'Bibo, Liana Veneziano, Barbara Garavaglia, Carlo Fusco, Nicholas Wood, Maria Stamelou, Nardo Nardocci

**Affiliations:** a Molecular Neurogenetics Unit, IRCCS Foundation Neurological Institute C. Besta, Via L. Temolo 4, 20126 Milan, Italy; b Department of Pediatric Neurology, IRCCS Foundation Neurological Institute C. Besta, Via Celoria 11, 20133 Milan, Italy; c Department of Medicine and Surgery, PhD Programme in Molecular and Translational Medicine, University of Milan Bicocca, Via Cadore 48, 20900 Monza, Italy; d Department of Molecular Neuroscience, UCL Institute of Neurology, WC1N 3BG London, United Kingdom; e Department of Neurology, Northwestern University, Feinberg School of Medicine, Chicago, 60611 Illinois, USA; f Child Neurology and Psychiatry Unit, Department of Pediatrics, IRCCS Santa Maria Nuova Hospital, Viale Risorgimento 80, 42123 Reggio nell’Emilia, Italy; g First Pediatric Clinic, University of Athens, Agia Sofia Children's Hospital, Thivon and Levadias, 11527 Athens, Greece; h Second Department of Neurology, Attiko University Hospital, Medical School, National and Kapodistrian University of Athens, Athens, Greece; i Third Department of Paediatrics, Attiko University Hospital, Medical School, National and Kapodistrian University of Athens, Athens, Athens, Greece; j Parkinson's Disease Center and Movement Disorders Clinic, Department of Neurology, Baylor College of Medicine, 7200 Cambridge, Houston, TX 77030-4202, USA; k Sobell Department of Motor Neuroscience and Movement Disorders, University College London, Institute of Neurology, London WC1N 3BG, United Kingdom; l Institute of Translational Pharmacology, National Research Council, Via Fosso del Cavaliere, 100, 00133 Rome, Italy; m Movement Disorders Department, HYGEIA Hospital, Athens, Greece

**Keywords:** *ADCY5*, Chorea, Dystonia, Myoclonus, Dyskinesia

## Abstract

**Introduction:**

*ADCY5* mutations have been recently identified as an important cause of early-onset hyperkinetic movement disorders. The phenotypic spectrum associated with mutations in this gene is expanding. However, the *ADCY5* mutational frequency in cohorts of paediatric patients with hyperkinetic movement disorders has not been evaluated.

**Methods:**

We performed a screening of the entire *ADCY5* coding sequence in 44 unrelated subjects with genetically undiagnosed childhood-onset hyperkinetic movement disorders, featuring chorea alone or in combination with myoclonus and dystonia. All patients had normal CSF analysis and brain imaging and were regularly followed-up in tertiary centers for paediatric movement disorders.

**Results:**

We identified five unrelated subjects with *ADCY5* mutations (11% of the cohort). Three carried the p. R418W mutation, one the p. R418Q and one the p. R418G mutation. Mutations arose *de novo* in four cases, while one patient inherited the mutation from his similarly affected father. All patients had delayed motor and/or language milestones with or without axial hypotonia and showed generalized chorea and dystonia, with prominent myoclonic jerks in one case. Episodic exacerbations of the baseline movement disorder were observed in most cases, being the first disease manifestation in two patients. The disease course was variable, from stability to spontaneous improvement during adolescence.

**Conclusion:**

Mutations in *ADCY5* are responsible for a hyperkinetic movement disorder that can be preceded by episodic attacks before the movement disorder becomes persistent and is frequently misdiagnosed as dyskinetic cerebral palsy. A residual degree of neck hypotonia and a myopathy-like facial appearance are frequently observed in patients with *ADCY5* mutations.

## Highlights

• *ADCY5* mutational frequency in paediatric patients is unknown.
• 5/44 (11%) subjects in our cohort carried pathogenic *ADCY5* mutations.
• Chorea-dystonia, exacerbations and developmental delay are often observed together.
• Disease course and clinical features are variable among patients with *ADCY5* mutations.
• Residual cervical hypotonia and a myopathy-like face are additional diagnostic clues.

## 1 Introduction

Adenyl cyclase 5, encoded by *ADCY5*, is a striatal-specific enzyme that converts adenosine triphosphate (ATP) into cyclic adenosine monophosphate (cAMP), an intracellular second messenger crucial for several molecular pathways [1].

The role of pathogenic mutations in *ADCY5* was first recognized in 2012, when a segregating missense change in the gene was discovered in a large dominant kindred with multiple affected members presenting with an early-onset hyperkinetic movement disorder named Familial Dyskinesia with Facial Myokymia (FDFM; OMIM 600293) [1], [2]. *A* second *de novo* mutation (p.R418W) in *ADCY5* was subsequently found in two unrelated patients presenting with childhood-onset chorea and dystonia [3] and mutation-positive subjects were also found in a cohort of patients with a clinical diagnosis of benign hereditary chorea (BHC) but no *NKX2-1* mutations [4]. The clinical phenotype associated with *ADCY5* mutations includes in most cases childhood-onset chorea with episodic exacerbations observed more frequently upon awakening, when falling asleep or during intercurrent illnesses [5], [6], [7], [8]. Besides chorea, various hyperkinetic movement disorders such as myoclonus and dystonia have been described in *ADCY5* positive subjects, but the prevalence of *ADCY5* mutations in such patients is unknown.

The aim of this study was to establish the contribution of *ADCY5* mutations in a multi-centric cohort of patients with early-onset hyperkinetic movement disorder who lacked a definite genetic diagnosis.

We identified six new European cases with pathogenic *ADCY5* mutations belonging to five different families, showing the clinical course of disease at different ages, phenotypic heterogeneity and variability of movement disorder.

## 2 Materials and methods

In this study, we included patients displaying paediatric onset hyperkinetic movement disorder featuring chorea alone or in combination with myoclonus and dystonia, including patients diagnosed with dyskinetic cerebral palsy (CP). Patients with secondary movement disorders, such as documented hypoxic injury at birth or with detectable structural brain lesions were not included. Patients enrolled had previously undergone extensive metabolic screening (plasma and urinary aminoacids and organic acids, lactate/pyruvate, cerebrospinal fluid analysis including neurotransmitters and biopterins dosage) and multiple MRI brain scans that were unrevealing. Mutations in the *NKX2-1* gene, a significant though rare cause of childhood-onset chorea, were excluded in all of these patients [9].

44 unrelated patients were included from five different European Centers (IRCCS C. Besta Neurological Institute, Milan; IRCCS Santa Maria Nuova Hospital, Reggio Emilia; Movement Disorders Department, HYGEIA Hospital, Athens; Second Department of Neurology, Attikon Hospital, University of Athens; First Paediatric Clinic, University of Athens, Agia Sofia Hospital, Athens). Details on clinical history were obtained by direct interviewing the patients and their relatives; in some cases, home-made videos were retrieved and reviewed by the authors to better define the clinical phenotype at earlier disease stages.

After obtaining informed consent (parental consent for minors where applicable), patients were blood sampled and DNA was extracted from peripheral blood lymphocytes according to standard procedures. *ADCY5* exons 2 and 10, in which mutations have been identified in most of the families published to date, were Sanger sequenced. Samples without mutations in these two exons were submitted for Whole Exome Sequencing (WES), which was performed as previously reported [10]. Segregation analysis in available family members was performed in all positive cases.

## 3 Results

Five out of 44 unrelated patients (11%) carried *ADCY5* mutations. Four patients were sporadic and carried *de novo* changes, while one had an autosomal dominant family history and inherited the mutation from his 47-year-old father, who also suffered from childhood-onset generalized chorea and dystonia. All mutations detected were located in *ADCY5* exon 2, at amino acidic residue 418 (p.R418W in 3 patients, p. R418G and p. R418Q in one each). Analysis of WES data did not reveal any additional mutation in *ADCY5* located outside exons 2 and 10 in the remainder 39 patients.

Clinical features of positive patients are summarized in Table 1.

**Table 1:** Clinical features of *ADCY5*-positive patients.

**Patient#**	***ADCY5*****mutation**	**Gender**	**Family history**	**AAO (MD)**	**Current age**	**Additional signs at onset**	**MD at onset**	**Current MD**	**Nocturnal paroxysms**	**Diurnal paroxysms**	**Paroxysmal episodes amelioration**	**Motor delay**	**Language delay**
**Pt 1**	c.1252C>T; p.R418W	F	N	1.5	15	Axial hypotonia	Paroxysmal dystonic episodes	Chorea, dystonia	Y	Y	Y	Y	Y
**Pt 2**	c.1253G>A; p.R418Q	M	N	1	18	Spastic gait	Paroxysmal dystonic episodes	Myoclonus, dystonia	Y	Y	Y	Y	N
**Pt 3**	c.1252C>G; p.R418G	M	Y	1	3	Axial hypotonia	Chorea, dystonia	Chorea, dystonia	N	N	NA	Y	Y
**Pt 4**	c.1252C>G; p.R418G	M	Y	3	47	UK	Chorea	Chorea, dystonia	Y	Y	N	Y	Y
**Pt 5**	c.1252C>T; p.R418W	F	N	3 mo	35	Axial hypotonia	Chorea	Chorea, dystonia	Y	Y	Y	Y	Y
**Pt 6**	c.1252C>T; p.R418W	M	N	2	5	Axial hypotonia	Chorea	Chorea, dystonia	N	Y	N	Y	Y

MD: movement disorder; AAO: age at onset; UK: unknown; NA: not applicable; mo: months.

**Patient 1** (p.R418W; *de novo* mutation) is a 15-year-old girl born pre-term from healthy parents. She presented with axial hypotonia (**Video 1 - Segment 1**) and delayed language. Around 11 months she developed abrupt brief generalized dystonic attacks when falling asleep. Between age 1 and 2, generalized chorea also appeared during attacks, that occurred in clusters on a weekly basis. Around 18 months of age she developed generalized chorea with a slowly progressive course until age 13 (**Video 1 - Segment 2**), and subsequent spontaneous improvement; at age 9 she developed left foot dystonia (in-turning). Due to chorea and severe axial hypotonia she could walk independently only at age 5; residual neck hypotonia is still present to date. Routine EEG and sleep studies did not show cortical correlates of movement disorder and brain MRI was unremarkable. She initially received a diagnosis of dyskinetic CP. On examination at age 15 (**Video 1 – Segment 3**), her mouth was slightly open, she showed generalized chorea involving also perioral muscles, dystonic posturing of upper and lower limbs, head drop and severe dysarthria with saliva drooling. Her total IQ (84) was in the borderline range (WISC). During teen age, episodic exacerbations of chorea and dystonia during sleep became shorter and less frequent and are now present about once a month. Episodic exacerbations also occur during the day with two distinct patterns: 1) sudden give-way of legs with falls to the ground with preserved consciousness and 2) generalized dystonic-choreic attacks favored by tiredness and narrow passages. Trihexyphenidyl up to 32 mg/day did not improve significantly motor symptoms.

Supplementary video related to this article can be found at http://dx.doi.org/10.1016/j.parkreldis.2017.05.004.

The following are the supplementary data related to this article:

Video1 segment 1 **Video 1 (Patient 1), Segment 1 (age 3):** severe developmental delay with axial and cervical hypotonia, tiptoe walking and generalized chorea; **(age 4)**: improvement of gait, persistence of cervical hypotonia and chorea. Video1 segment 1

Video1 segment 2 **Segment 2 (age 9)**: generalized chorea, left foot dystonia (inturning), dystonic posturing of upper limbs when outstretched. Video1 segment 2

Video1 segment 3 **Segment 3 (age 15)**: generalized chorea involving also facial muscles, myopathy-like face with mouth kept open, dysarthria, residual cervical hypotonia (neck flexion). Video1 segment 3

**Patient 2** (p.R418Q; *de novo* mutation) is an Italian 18-year-old boy born from healthy parents. He presented with delayed motor milestones and a tendency to tiptoe walking at 18 months. Since 6 months of age, nocturnal attacks of generalized dystonia with inconsolable crying, lasting up to some hours, disrupted his sleep. During infancy he developed generalized chorea and mild myoclonic jerks also involving facial muscles (**Video 2 – Segment 1**) and he showed a mildly scissoring gait with pyramidal signs in the lower limbs, for which he underwent tendon elongation. Episodic worsening of dyskinesias accompanied by hyperventilation, lasting about half an hour, were noticed during childhood, triggered by emotions and stress; sometimes hyperventilation and tachypnoea occurred without exacerbation of dyskinesias. These episodes initially recurred at weekly intervals and then spontaneously decreased over disease course. Currently, diurnal and nocturnal exacerbations are almost abolished (about one episode a year is reported), the most recent one being triggered by a minor orthopedic injury. Chorea slowly improved during teen-age years, and the clinical picture became dominated by dystonia and myoclonus. On examination at age 17, he showed mildly scissoring gait with pyramidal signs in the lower limbs, mild dysarthria with a tendency to keep his mouth open, cervical dystonia, multifocal non-stimulus sensitive myoclonic jerks at rest and on posture more prominent in the upper body, also involving the perioral muscles (**Video 2** - **Segment 2**). Standard EEG and sleep studies showed no EEG correlates of hyperkinesias. EMG recordings revealed bursts of 100–120 ms in the upper limbs and neck, alone or superimposed on dystonic co-contraction of antagonistic muscle groups, consistent with the co-occurrence of myoclonus and dystonia, as observed in DYT11 positive patients [11]. Clonazepam was not beneficial in reducing myoclonus. The patient's IQ (WISC) at age 7 was 86.

Supplementary video related to this article can be found at http://dx.doi.org/10.1016/j.parkreldis.2017.05.004.

The following are the supplementary data related to this article:

Video 2 segment 1 **Video 2 (Patient 2), Segment 1: (age 7 and 8)**: generalized chorea with facial involvement and superimposed myoclonic jerks, more severe in the upper limbs; cervical hypotonia, myopathy-like face. Video 2 segment 1

Video 2 segmeny 2 **Segment 2 (age 17)**: mildly scissoring gait, multifocal myoclonic jerks, cervical dystonia (left torticollis) and right upper limb posturing; chorea involving perioral muscles, myopathy-like face with mouth kept open. Video 2 segmeny 2

**Patient 3** (p.R418G; inherited mutation) is a 3-year-old Italian boy born full-term after normal pregnancy. Motor and language development were delayed, he managed to sit unsupported at 17 months and to walk unassisted at 2 years of age. Generalized chorea appeared in the first months of life. Neurological examination at age 2.5 showed axial hypotonia, mild generalized chorea and dystonic posturing of the limbs (tiptoe walking). To date, the patient has not presented diurnal or nocturnal paroxysmal exacerbations of chorea. His father (**Patient 4**), 47 years old, had delayed motor and language milestones. Generalized chorea with dystonic posturing of upper limbs appeared around age 3. Since childhood, he has suffered from severe and painful exacerbations of dyskinesias triggered by emotions and tiredness and also present during sleep. Chorea is currently worsened by action, emotions and stress (**Video 3**). Acetazolamide significantly improved its severity, whereas tetrabenazine, baclofen and trihexyphenidyl were not effective. His son was started on acetazolamide (125 mg/day) with no substantial changes in his mild movement disorder.

Supplementary video related to this article can be found at http://dx.doi.org/10.1016/j.parkreldis.2017.05.004.

The following is the supplementary data related to this article:

Video 3 **Video 3 (Patient 4, age 47):** generalized chorea also involving facial muscles; myopathy-like face with mouth kept open. Video 3

**Fig. 1:**
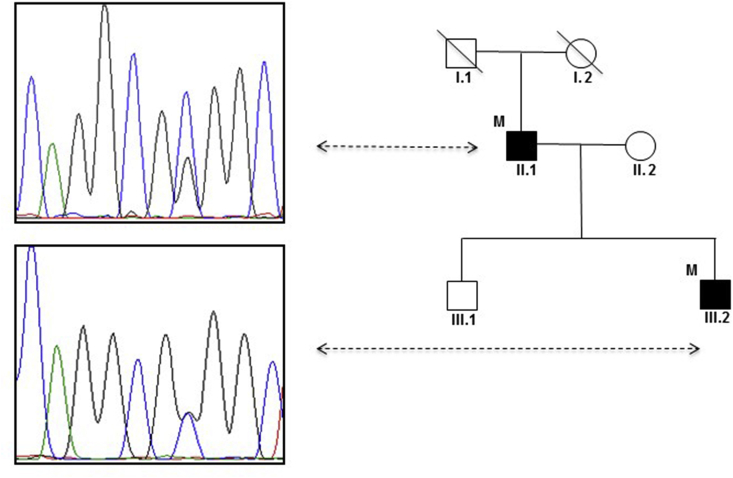
Pedigree of patients 3 and 4. The electropherogram of Patient 4 (II.1) shows an unbalanced ratio between the wild-type and the mutant allele (top panel), with the wild-type significantly more represented, suggesting somatic mosaicism. This was not observed in Patient 3 (III.2), where the chromatograms of the normal and mutated allele are equivalently represented (bottom panel).

Both the patient and his father carried the p. R418G change; visual inspection of the chromatograms showed an imbalanced ratio between the wild-type and the mutated allele, with the latter significantly less represented (Fig. 1). This was not observed in the son, suggesting that the father could be a mosaic for the mutation.

**Patient 5** (p.R418W; *de novo* mutation) is a 35-year-old woman of Greek origin, born full term after an uneventful pregnancy. At 3 months of age she had failure to thrive, feeding difficulties, and developed choreic movements. At the age of 9 months she could not sit unsupported and showed axial hypotonia. At age two, she had a mild cognitive and motor developmental delay. She initially received a diagnosis of dyskinetic CP, with normal intelligence. Her clinical picture remained stable until age 7, when she developed sudden attacks characterized by hip and trunk flexion that made her collapse to the ground with no alteration of consciousness (**Video 4 – Segment 1**). She also developed sustained dystonic postures of the limbs both throughout the day and night, particularly upon awakening, which at times were extremely painful. Multiple brain MRI scans and EEGs were normal. Over the following years, chorea remained stable and the paroxysmal episodes had a variable course, with spontaneous remission for about two years and reappearance at age 23 after a traumatic event. The episodes lasted up to 30 min and could occur many times a day and were diagnosed as functional (psychogenic). On examination at age 35 (**Video 4 – Segment 2**), her mouth was slightly open and she had some drooling, and a dysarthric speech. Chorea was present in the face, involving mainly the mouth, and limbs were mildly affected as well, especially the arms. There was dystonic posturing of the feet and hands when outstretched. She was hypotonic, reflexes were present and symmetric throughout. Sleep studies showed hypoventilation triggering paroxysmal episodes at night, for which she was given a CPAP treatment with some improvement of the nocturnal episodes. Tetrabenazine, levodopa, trihexyphenidyl, various anticonvulsants were not helpful. Clonazepam significantly improved the frequency and severity of the paroxysmal episodes. The patient was found to carry a *de novo* p. R418W mutation.

Supplementary video related to this article can be found at http://dx.doi.org/10.1016/j.parkreldis.2017.05.004.

The following is the supplementary data related to this article:

Video 4 Segment 1 and 2 **Video 4 (Patient 5), segment 1 (age 10)**: severe generalized chorea and axial hypotonia; **age 17**: episodic falls to ground; **segment 2 (age 35)**: facial grimacing, distal chorea of upper and lower limbs. Video 4 Segment 1 and 2

**Patient 6** (p.R418W; *de novo* mutation) is a 5 year-old Greek child born full term from healthy parents. He had delayed milestones and generalized hypotonia and was able to sit unassisted and to stand with support only at 20 months of age. He also had delayed language development, though verbal understanding was good. Generalized chorea appeared around age one, and about one year later he developed brief diurnal paroxysmal events lasting less than one minute, that were characterized by limb posturing, more severe in the arms, associated with possible axial posturing. Brain MRI and CSF analysis were unremarkable. On examination at age 5, he was able to follow simple commands and had moderate generalized chorea involving also the face, along with generalized hypotonia. Paroxysmal dystonic attacks are still present, especially upon awakening. A trial of Levodopa was not effective and no additional medication was started.

## 4 Discussion

Since the original report in 2001 [2], in which the authors described the movement disorder in the affected family members as “Familial Dyskinesia with Facial Myokymia”, the phenotypic spectrum associated with *ADCY5* mutations has expanded and a more detailed delineation of movement disorders has been provided in subsequent reports [3], [4], [5], [7].

So far, 60 genetically confirmed patients (24 sporadic, 36 familial cases) belonging to 36 different families have been reported (Table 2). The mutational frequency of *ADCY5* in homogeneous cohorts of patients with early-onset non-progressive hyperkinetic movement disorders has not been assessed in previous publications, and some authors identified positive subjects in extensive screenings of patients affected by heterogeneous movement disorders [12]. In this study, we aimed at defining the contribution of *ADCY5* mutations in a cohort of patients with a childhood-onset hyperkinetic movement disorder. We found that 11% of our cohort carried *ADCY5* pathogenic mutations. These patients displayed most of the previously described features of *ADCY5*-associated disease: (a) onset in infancy-childhood with delayed milestones and axial hypotonia, (b) a mixed hyperkinetic movement disorder mostly characterized by generalized chorea and dystonia and (c) frequent exacerbations of dyskinesias upon awakening and when falling asleep. Importantly, differently from subjects with *ADCY5* mutations, these core features were not observed all together in any of the 39 subjects without mutations, suggesting that their concomitant presence is a strong predictor of the mutational status.

**Table 2:** *ADCY5* positive patients and kindred reported in the literature to date.

**Publication**	***ADCY5*****positive patients**	**Affected subjects reported**^a^	**No of kindred**	***ADCY5*****mutation** (no of kindred, no of positive patients)
Chen et al., 2012 [1]	10	18	1	p.A726T
Chen et al., 2014 [3]	2	2	2	p.R418W
Chen et al., 2015 [5]	24	30	15	p.R418W (8 K, 9) p.R418Q (3 K, 3) p.A726T (1 K, 6) p.L720P (1 K, 1) p.R438P (1 K, 1) p.M1029K (1K, 4)
Mencacci et al., 2015 [4]	3	3	2	p.R418W
Carapito et al., 2015 [6]	2	2	1	c.2088+1G > A
Chang et al., 2016 [7]	6	10	6	p.R418W (4 K, 4) p.R418G (1 K, 1) p.R418Q (1 K, 1)
Dy et al., 2016 [8]	3	4	3	p.R418W (2 K, 2) p.K694_M696 (1 K, 1)
Zech et al., 2016 [12]	3	3	2	p.I460F (1K, 1) p.R727K (1K, 2)
Meijer et al., 2016 [15]	1	1	1	p.R418W
Westenberger et al., 2016 [17]	2	2	2	p.D1015E (1K, 1) p.E1025V (1K, 1)
Douglas et al., 2017 [18]	4	5	1	p. M1029R
**TOTAL**	**60**	**80**	**36**	

K: kindred.

a Including clinically affected subjects lacking genetic confirmation.

Some patients were previously diagnosed with dyskinetic CP despite normal MRI findings and no clear perinatal injury. While movement disorders in CP can show significant worsening during intercurrent infections, a clear relationship between the exacerbations of movement disorders and sleep should raise the suspicion of a misdiagnosis and lead to consider underlying *ADCY5* mutations.

Chorea presenting in infancy is often due to acute basal ganglia damage in the context of metabolic encephalopathies (aminoacidopathies, organic acidurias, Lesh-Nyhan syndrome), while in childhood auto-immune causes with normal brain imaging prevail (Sydenham's chorea, autoimmune encephalitides) and rare non-metabolic genetic conditions must also be considered (*NKX2-1*, *PDE10A*, *GNAO1* mutations) [13].

Age at onset of paroxysmal dyskinesias and dystonia ranged between 6 months and seven years of age in our study, being the first disease manifestation (along with delayed milestones) in one third of positive patients, and developing before a chronic movement disorder became evident. Onset in the first months of life and relation to sleep is unusual in paroxysmal movement disorders due to *PRRT2*, *PNKD* and *SLC2A1* mutations, thus mutations in *ADCY5* should be considered in the differential of paroxysmal movement disorders with a very early onset even in the absence of a detectable chronic movement disorder. On the other hand, as exemplified by Patient 3, who displays mild chorea and dystonia without paroxysmal exacerbations, the absence of such manifestations, suggested as a “red flag” for *ADCY5*-related dyskinesias [14], does not rule *ADCY5* mutations out, at least in the first years of life.

The natural history of *ADCY5*-related dyskinesias is still poorly defined and no or little progression of movement disorder has been observed in previous reports. Phenotypic variability has been partly attributed to genotype-phenotype correlation, with available evidence suggesting that the A726T mutation is associated with a milder phenotype [5], whereas the p. R418W is responsible for a more severe clinical picture. A lesser degree of severity of movement disorder has also been explained with somatic mosaicism [4], [5]. In our series, chromatograms of Patient 4 (Fig. 1) were suggestive of somatic mosaicism; however, he displayed a relatively severe phenotype as compared to his son. This might indicate that additional genetic and/or environmental factors may play a role in determining *ADCY5* phenotypic variability.

In our series, patients were regularly assessed for several years, thus disease course and phenomenology could be documented at different ages. All patients presented with delayed ability to sit unsupported or walk independently and showed a combination of axial hypotonia and chorea/dystonia affecting also the lower limbs together with spasticity in one case (Patient 2). Axial hypotonia slowly improved over disease course, but a residual degree of cervical hypotonia could still be observed in adolescence in some affected subjects (Patient 1 and 2). Cervical hypotonia in adolescents and young adults can mimic dystonic anterocollis, and it is detectable in other positive patients from previously published videos [7], thus representing a potential additional clue to individuate *ADCY5* mutation carriers, as observed by Meijer et al. [15]. Abrupt violent head drops are considered characteristic of chorea-acanthocytosis [16], but are phenomenologically different from what has been observed in *ADCY5* cases.

The course of dyskinesia exacerbations was variable in our series, including spontaneous amelioration in frequency and severity (Patient 1), almost complete remission during teen age (Patient 2), stability since onset (Patient 4 and 6) and stability with relatively long attack-free periods (Patient 5). Peripheral trauma was reported in two cases to trigger recrudescence of attacks after free periods.

Movement disorder disease course was variable as well, with spontaneous improvement of chorea observed in some cases during adolescence and relative stability since childhood in others.

Patient 2 switched from a choreic/dystonic phenotype in childhood to a clinical and electrophysiological picture consistent with myoclonus-dystonia in his late teens, and was in fact previously tested for DYT11 mutations; however, the presence of pyramidal signs in the lower limbs and delayed milestones in infancy were not consistent with the classic myoclonus-dystonia phenotype due to DYT11 mutations.

In terms of treatment, several agents such as tetrabenazine, trihexyphenidyl, levodopa and anticonvulsant were not beneficial. Of note, acetazolamide, a carbonic anhydrase inhibitor, significantly improved chorea in Patient 4; response of dyskinesia to this drug, though considered nonspecific, was reported in two patients of the original family published by Fernandez et al. [2]. Clonazepam reduced the dystonic episodes in Patient 5 but was ineffective in ameliorating myoclonus in Patient 2. Given the unsatisfactory response of *ADCY5*-related movement disorders to several drugs, bilateral GPi Deep Brain Stimulation (DBS) has been recently performed in four patients, with moderate reduction of hyperkinetic movements [8], [15].

In our series, all positive patients carried mutations involving the arginine 418, and half of them carried the p. R418W mutation, which is by far the most frequently encountered missense variant, being present in 19/36 (53%) unrelated probands reported to date. With our series, the total number of *ADCY5* positive patients reaches 66 cases and the number of unrelated patients carrying the p. R418W mutation increases up to 22/41 (54%) (Table 3). We therefore confirm that arginine 418 is a mutational hot spot in *ADCY5* with a relevant pathogenic role.

**Table 3:** Frequency of *ADCY5* mutations reported.

**ADCY5 mutation**	**Affected cases reported (%)**^a^	**Number of kindred reported (%)**^a^
c.1252C > T; p.R418W	24 (36,4%)	22 (54%)
c.2176G > A; p.A726T	16 (24,2%)	2 (4,9%)
c.1253G > A; p.R418Q	5 (7,6%)	5 (12.2%)
c.3086T > A; p.M1029K	4 (6%)	1 (2,4%)
c.3086T > G; p. M1029R	4 (6%)	1 (2,4%)
c.1252C > G; p.R418G	3 (4,5%)	2 (4,9%)
c.2088+1G > A	2 (3%)	1 (2,4%)
c.2180G > A; p.R727K	2 (3%)	1 (2,4%)
c.2159T > C; p.L720P	1 (1,5%)	1 (2,4%)
c.1313G > C; p.R438P	1 (1,5%)	1 (2,4%)
c.2080_2088del; p.K694_M696	1 (1,5%)	1 (2,4%)
c.3045C > A; p.D1015E	1 (1,5%)	1 (2,4%)
c.3074A > T; p.E1025V	1 (1,5%)	1 (2,4%)
c.1378A > T; p.I460F	1 (1,5%)	1 (2,4%)
**Total**	**66 (100%)**	**41 (100%)**

a Including the present paper.

## 5 Conclusions

Mutations in *ADCY5* represent a significant genetic cause of early-onset non-progressive hyperkinetic movement disorders, with a frequency of 11% in our series. The increasing number of cases reported is contributing to define the phenotypic spectrum of this disorder. Delayed milestones and axial hypotonia seem to be almost universal features in infancy, while onset of movement disorder and its episodic or chronic nature are variable in the first disease phases and tend sometimes to spontaneously improve with age. We suggest testing for *ADCY5* mutations patients previously diagnosed with dyskinetic cerebral palsy when exacerbations of dyskinesia are clearly sleep-related. The knowledge about long-term motor outcome in affected children is still limited, given the relatively small number of cases reported so far. We observed some common features in most patients, including the presence of “head drop” probably due to residual cervical hypotonia as well as a myopathy-like appearance of face with mouth kept slightly open. These characteristics may be relevant in patients without frequent episodic movement disorder exacerbations and suggest underlying *ADCY5* mutations, although their pathophysiology still needs to be elucidated.

## Author roles

Miryam Carecchio: concept and design, data collection, data analysis, drafting of manuscript, manuscript revision; Niccolò E. Mencacci: concept and design, data collection, data analysis, manuscript revision; Alessandro Iodice: data collection, data analysis; Celeste Panteghini: data collection, data analysis, manuscript revision; Roser Pons: data collection, manuscript revision; Giovanna Zorzi: data collection; Federica Zibordi: data collection; Anastasios Bonakis^:^ data collection; Argyris Dinopoulos: data collection; Joseph Jankovic: data collection, manuscript revision; Leonidas Stefanis: data collection; Kailash P. Bhatia: data collection, manuscript revision; Valentina Monti: data collection; Lea R'Bibo: data collection, data analysis; Barbara Garavaglia: manuscript revision; Nicholas Wood: data collection; Carlo Fusco: data collection, manuscript revision; Maria Stamelou: concept and design, data collection, manuscript revision; Nardo Nardocci: manuscript revision.

## Fundings

This work received financial support from the Fondazione Pierfranco e Luisa Mariani. This work was supported financially by a Medical Research Council/Wellcome Trust Strategic Award (WT089698/Z/09/Z) and grants from the Bachman-Strauss Dystonia Parkinsonism Foundation, and NIHR Bioresource Rare Diseases. The funders had no role in study design, data collection and analysis, decision to publish, or preparation of the manuscript. The work was undertaken at University College London (UCL), who receive support from the Department of Health's National Institute for Health Research (NIHR) Biomedical Research Centers funding streams. N.E.M. is funded by a NIHR funding scheme.

## Financial disclosures/conflict of interest

None to declare.
