# Supplementary figures and images for: ADCY5-related movement disorders: Frequency, disease course and phenotypic variability in a cohort of paediatric patients
Source: Parkinsonism Relat Disord. 2017 Aug;41:37–43. doi: 10.1016/j.parkreldis.2017.05.004 (PMC5549507; doi:10.1016/j.parkreldis.2017.05.004)

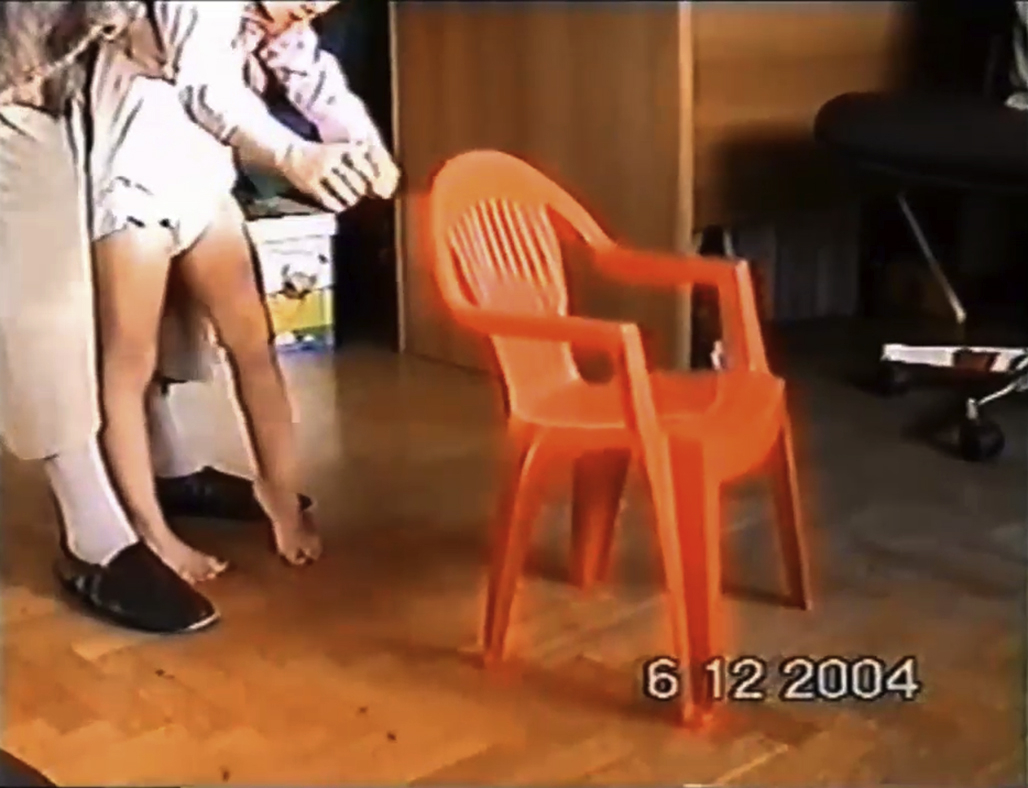

Supplement: Video1 segment 1 — Video 1 (Patient 1), Segment 1 (age 3): severe developmental delay with axial and cervical hypotonia, tiptoe walking and generalized chorea; (age 4): improvement of gait, persistence of cervical hypotonia and chorea. [file mmc1.jpg]

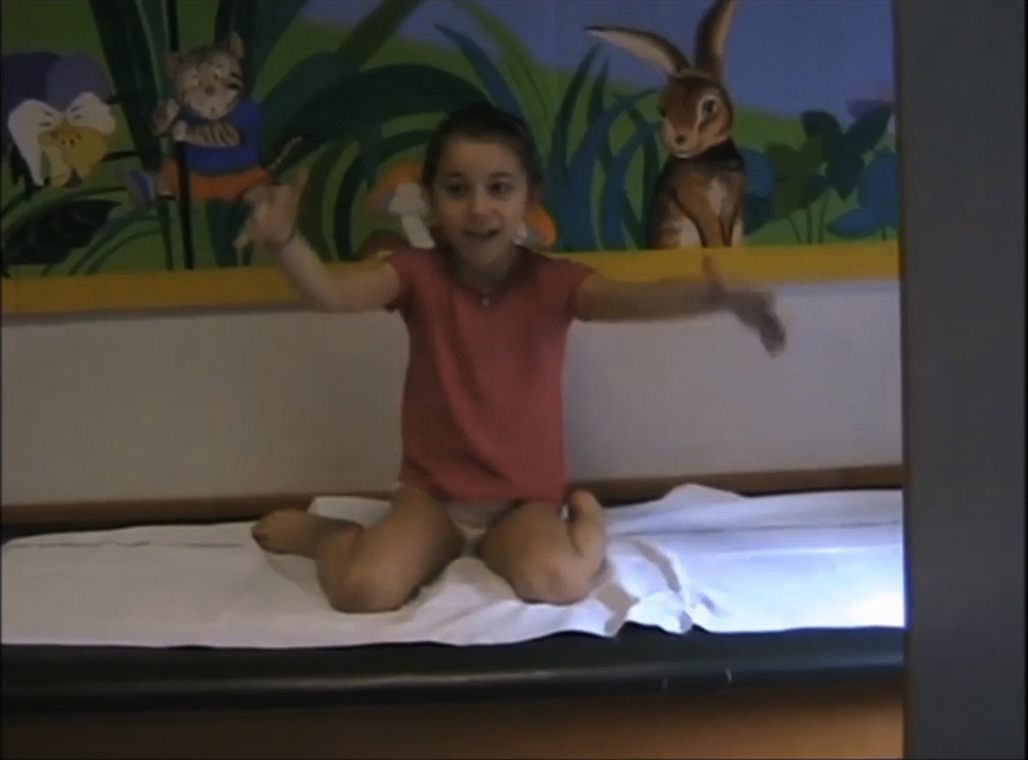

Supplement: Video1 segment 2 — Segment 2 (age 9): generalized chorea, left foot dystonia (inturning), dystonic posturing of upper limbs when outstretched. [file mmc2.jpg]

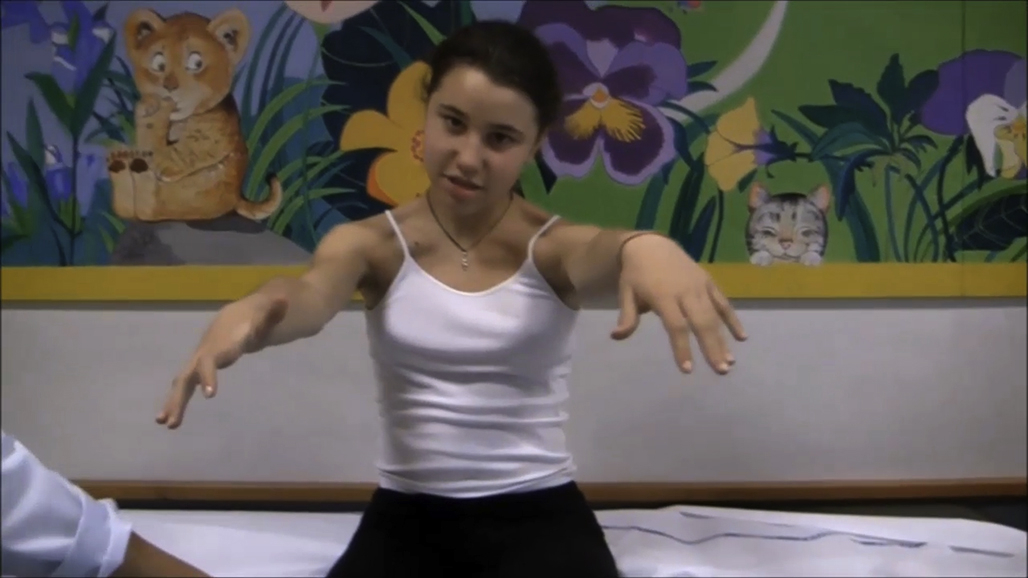

Supplement: Video1 segment 3 — Segment 3 (age 15): generalized chorea involving also facial muscles, myopathy-like face with mouth kept open, dysarthria, residual cervical hypotonia (neck flexion). [file mmc3.jpg]

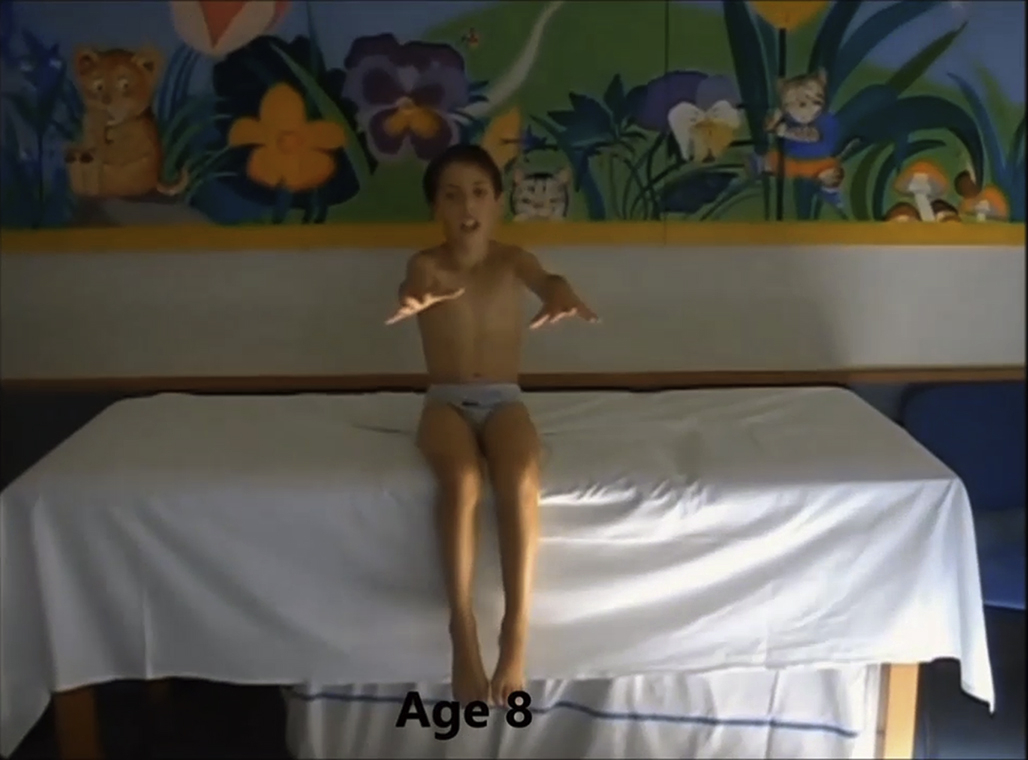

Supplement: Video 2 segment 1 — Video 2 (Patient 2), Segment 1: (age 7 and 8): generalized chorea with facial involvement and superimposed myoclonic jerks, more severe in the upper limbs; cervical hypotonia, myopathy-like face. [file mmc4.jpg]

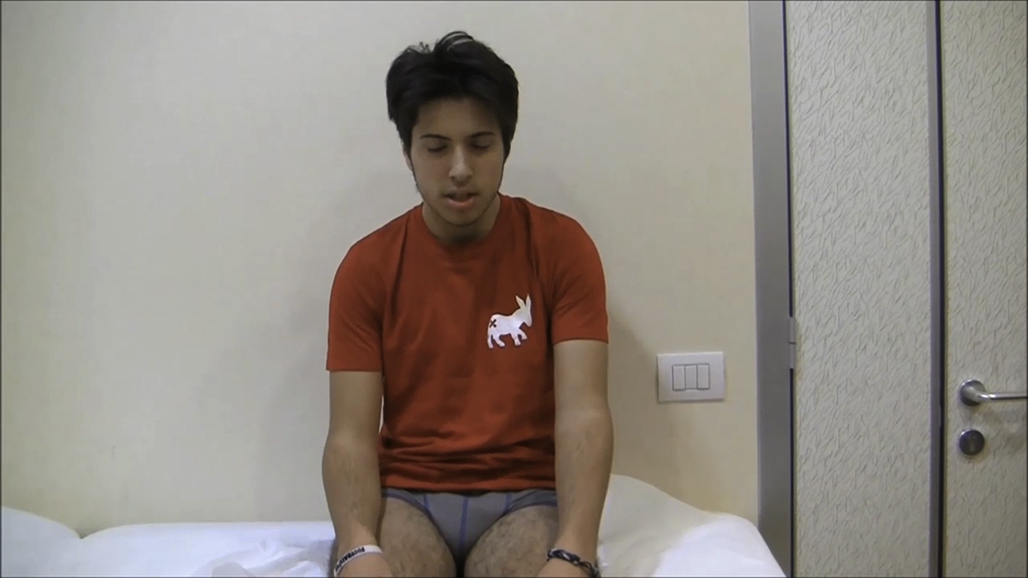

Supplement: Video 2 segmeny 2 — Segment 2 (age 17): mildly scissoring gait, multifocal myoclonic jerks, cervical dystonia (left torticollis) and right upper limb posturing; chorea involving perioral muscles, myopathy-like face with mouth kept open. [file mmc5.jpg]

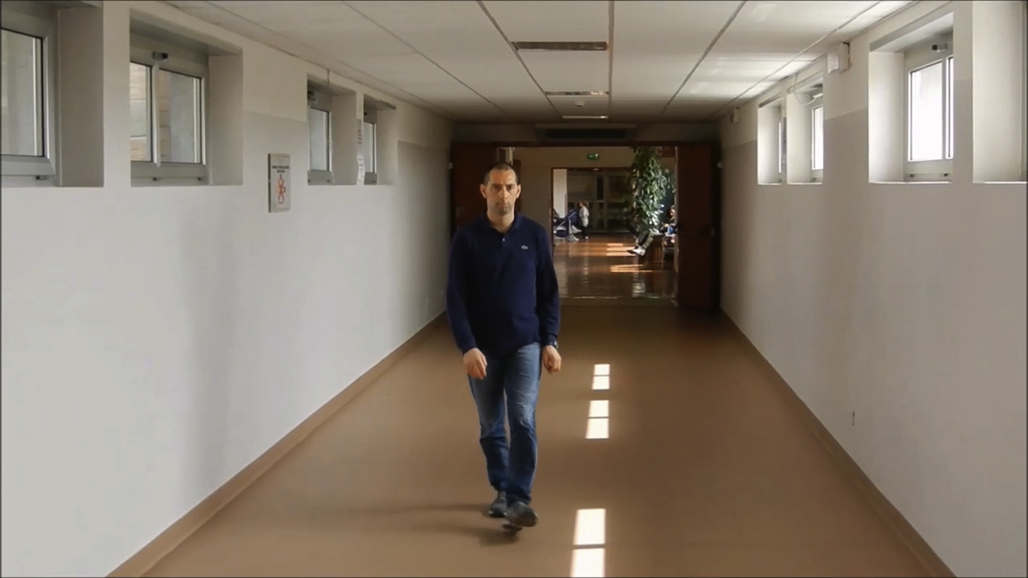

Supplement: Video 3 — (Patient 4, age 47): generalized chorea also involving facial muscles; myopathy-like face with mouth kept open. [file mmc6.jpg]

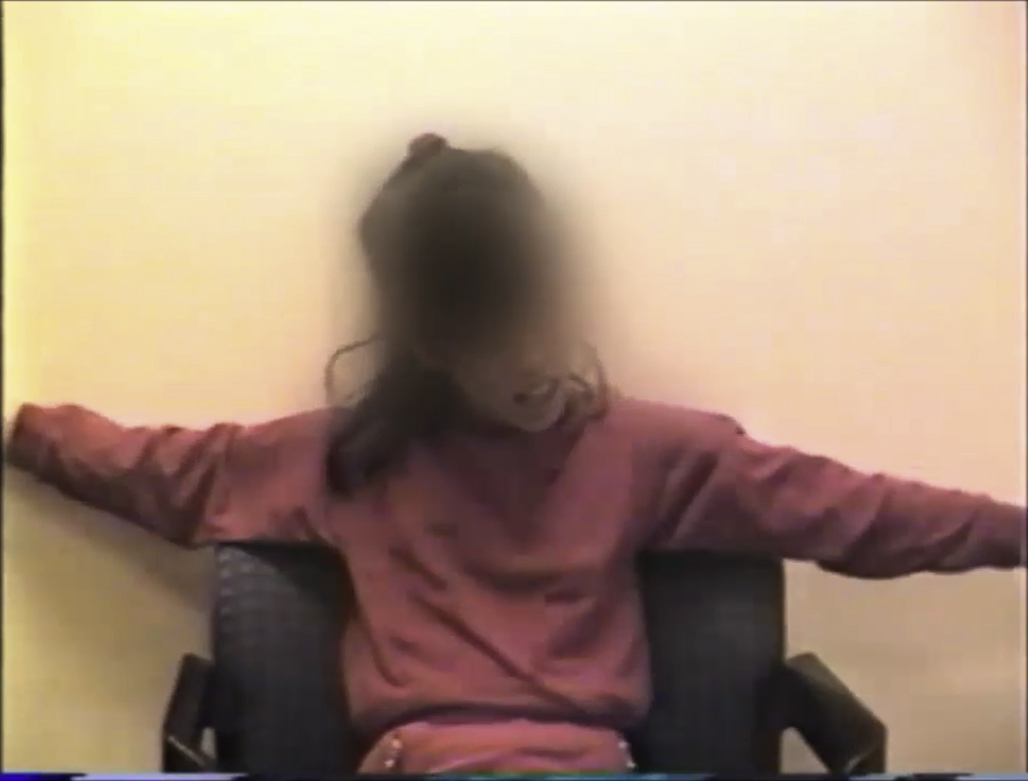

Supplement: Video 4 Segment 1 and 2 — Video 4 (Patient 5), segment 1 (age 10): severe generalized chorea and axial hypotonia; age 17: episodic falls to ground; segment 2 (age 35): facial grimacing, distal chorea of upper and lower limbs. [file mmc7.jpg]
